# Myoclonus generators in sialidosis

**DOI:** 10.1016/j.cnp.2022.05.004

**Published:** 2022-06-10

**Authors:** Felipe Vial, Patrick McGurrin, Sanaz Attaripour, Alesandra d'Azzo, Cynthia J. Tifft, Camilo Toro, Mark Hallett

**Affiliations:** a Human Motor Control Section, National Institute of Neurological Disorders and Stroke, National Institutes of Health, Bethesda, MD, USA; b Facultad de Medicina Clínica Alemana Universidad del Desarrollo, Santiago, Chile; c Department of Genetics, St. Jude Children’s Research Hospital, Memphis, TN, USA; d Undiagnosed Diseases Program, National Institutes of Health, Bethesda, MD, USA; e Medical Neurology Branch, National Human Genome Institute, Bethesda, MD, USA; f Department of Neurology, University of California, Irvine, 200 S. Manchester Ave., Ste 206, Orange, CA 92868, USA

**Keywords:** Sialidosis, Myoclonus

## Abstract

**Objective:**

Sialidosis is an inborn error of metabolism. There is evidence that the myoclonic movements observed in this disorder have a cortical origin, but this mechanism does not fully explain the bilaterally synchronous myoclonus activity frequently observed in many patients. We present evidence of a subcortical basis for synchronous myoclonic phenomena.

**Methods:**

Electromyographic investigations were undertaken in two molecularly and biochemically confirmed patients with sialidosis type-1.

**Results:**

The EMG recordings showed clear episodes of bilaterally synchronous myoclonic activity in contralateral homologous muscles. We also observed a high muscular-muscular coherence with near-zero time-lag between these muscles.

**Conclusion:**

The absence of coherence phase lag between the right-and-left homologous muscles during synchronous events indicates that a unilateral cortical source cannot fully explain the myoclonic activity. There must exist a subcortical mechanism for bilateral synchronization accounting for this phenomenon.

**Significance:**

Understanding this mechanism may illuminate cortical-subcortical relationships in myoclonus.

## Highlights

• The cortical origin of myoclonus in sialidosis does not fully explain the phenomena.
• We used electrophysiology to show a possible subcortical source for the myoclonus.
• Correct understanding of this physiopathology may help improve treatment.

## 1 Introduction

Sialidosis is an autosomal recessive inborn error of metabolism caused by mutations in the *NEU1* gene, which encodes the lysosomal enzyme neuraminidase 1 (NEU1) (Oheda et al., 2006, D’Azzo et al., 2015). Patients are classified in two main subtypes, Sialidosis type-1, characterized by onset of progressively disabling myoclonus, seizures and macular degeneration (cherry-red spot) in the second decade of life, sometimes referred to as “cherry-red spot myoclonus syndrome” (Malek et al., 2015), and Sialidosis type-2, with an earlier and more severe course, which also includes skeletal deformities, hearing loss, hepatomegaly, dysmorphic features, and severe neurological involvement. The phenotypic differences may relate, at least in part, to the amount of residual NEU1 enzyme activity (Franceschetti and Canafoglia, 2016).

Regarding the generators of myoclonus in sialidosis, there is evidence that points to a cortical origin, mainly the cortical hyperexcitability and electroencephalographic (EEG) correlation of a cortical event preceding the onset of myoclonus (Franceschetti and Canafoglia, 2016). However, our observations, and those of other groups (Avanzini et al., 2016, Canafoglia et al., 2011), indicate that apart from the multifocal myoclonus, these patients often exhibit myoclonic activity consisting of bilateral synchronous activation of homologous muscle groups in the upper or lower extremities. This pattern is more compatible with a subcortical origin of the myoclonus. There is evidence, based on the comparison between the C reflex and the myoclonic latency, that patients with other forms of progressive myoclonic epilepsy may also have a subcortical source (Cantello et al., 1997).

Here we explore electrophysiologically the hypothesis for a subcortical generator accounting for the synchronous phenomena in two patients with sialidosis type-1.

## 2 Methods

### 2.1 Subjects

Two biochemically, molecularly, and clinically confirmed patients with Sialidosis type-1, 30 (female) and 41 (male) years of age, participated in the study (see Table 1 for clinical characteristics). Subjects gave their written informed consent for the Nervous System Degeneration in Glycosphingolipid Storage Disorders protocol (NCT00029965), approved by the NIH institutional review board.

**Table 1:** Clinical characteristics of the two patients.

**Patient**	1	2
**Age at time of study**	41	30
**Age of first symptom**	16	11
**First symptom**	Balance difficulty	Seizure
**Mental Exam**	Normal	Language Problems
**Cherry Spot**	Yes	Yes
**Ocular Movements**	Downbeat Nystagmus, slow horizontal saccades	Normal
**Tone**	Normal	Normal
**Strength**	4/5 globally	Normal
**Tendon Reflexes**	Brisk, symmetric	Brisk, symmetric
**Sensory Exam**	Normal	Mild loss of vibratory sensation in lower extremities
**Medication**	Zonisamide, Clonazepam	Levetiravetam, Valproic Acid, Piracetam, Trazodone, Acetazolamide

### 2.2 Experimental conditions

We used surface electromyography (EMG) and electroencephalography (EEG) to record muscle and brain activity, respectively, during all behavioral conditions. EEG and EMG were recorded using BrainVision recorder (BrainVision, Morrisville, NC) at a sampling rate of 5 kHz. EEG was recorded by a 64-channel Acticap system. We placed the ground at FPz and EEG recordings were referenced to the left mastoid. Impedances were kept below 10kΩ. Multichannel EMG data, as indicated in Table 2, were recorded using neonatal surface electrodes (3 M, Cardinal Health) in a bipolar montage.

**Table 2:** Electromyography targets during each experimental condition.

**Rest (recorded on more affected side)**	**Action (recorded bilaterally)**
Orbicularis Oculi	Biceps
Masseter	Triceps
Mentalis	Extensor Carpi Radialis
Sternocleidomastoid	Flexor Carpi Radialis

### 2.3 Behavioral testing

Rest Condition: Patients sat on a comfortable chair and were instructed to relax. Then we recorded two sets of EEG-EMG for 60 s.

Action Conditions: We recorded action myoclonus with EMG on proximal and distal upper extremities. The recordings included a) outstretched left arm, b) outstretched right arm, c) both arms outstretched, d) left finger to nose movement, e) right finger to nose movement. Two runs of 60 s of each condition were recorded.

### 2.4 Data preprocessing

Continuous EMG data were processed using Fieldtrip toolbox in Matlab (Oostenveld et al., 2011). Data were first band-pass filtered at 20–300 Hz for EMG. A notch filter (55–65 Hz) was also used to reduce AC line noise artifact.

After filtering, all channels were visually inspected. Those channels with too much noise resulting from faulty electrodes were removed from the analyses.

### 2.5 Data analysis

Continuous EMG data were visually inspected for the myoclonus characterization. For the back-averaging, the rest and action myoclonus were analyzed separately. For the action myoclonus all the conditions were amalgamated and analyzed together. We used a customized algorithm to look for and mark myoclonic bursts in each muscle (see Vial et al., 2020). The original data were then segmented according to the markers and averaged.

For the action myoclonus data, we compared the timing between muscle bursts in contralateral homologous muscles looking for bilateral bursts happening within a window of −20 to +20 ms range from each other. Then the distribution was calculated by clustering the latencies in 1 ms bins. To look for significance, a permutation test was done by segmenting the 10 min of action myoclonus data into 2-second epochs, randomly permutating the epochs 500 times, and re-calculating the distribution of latencies. The 95% limit was extracted from the distribution of the permutated data.

For muscular-muscular coherence, a wavelet transformation was done using a family of 150 wavelets linearly spaced from 1 to 150 Hz with a range of cycles between 4 and 10. After that, the magnitude squared coherence was calculated as the squared cross-spectral density, divided by the auto spectral density of each signal. The 95% significance was calculated according to the Halliday method (Halliday et al., 1995). Also, the phase difference was extracted and transformed to time lag for the segments with significant coherence.

## 3 Results

### 3.1 Myoclonic burst characterization

**Fig. 1:**
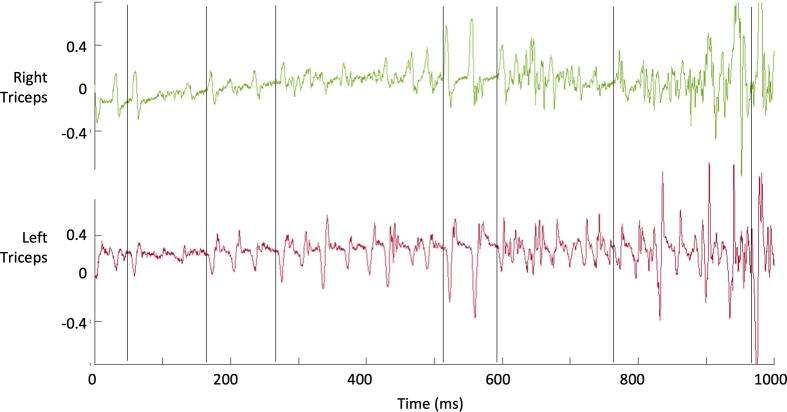
Electromyography example of a one second epoch from patient 1 holding both arms up. The vertical black lines highlight moments in which left and right sides are bursting together.

In the 2 cases, there was a pattern of 15–20 ms muscle bursts present mainly during posture and action. The pattern of bursting changed from time to time, but when present was rhythmic, with a frequency around 20 Hz. Most of the time, it was possible to observe synchronous activation of pairs of antagonist muscles. There were also episodes of evident synchronous activation of the homologous contralateral muscles (Fig. 1).

### 3.2 Back-averaging

**Fig. 2:**
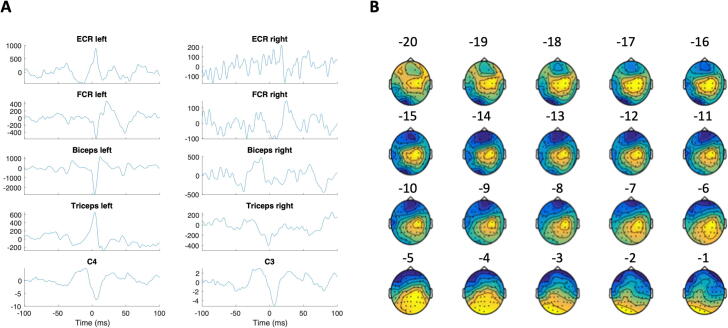
A: Mean traces from patient 1 after epoching data and back-averaging 3,302 movements initiating from the left biceps. There is agonist antagonist co-contraction of the left biceps/triceps. In addition, there is later contraction of both ipsilateral distal muscles, flexor and extensor carpi radialis, and contralateral muscles. There is also a clear electroencephalographic potential preceding the burst. B: Topographic representation of the electroencephalographic potential preceding the muscle burst from −20 ms to −1 ms. The potential is localized to the contralateral motor cortex.

The back average clearly shows an EEG potential preceding the burst, most of the time localizing to the contralateral motor cortex (Fig. 2). The rhythmicity of the bursting is also clearly seen in both the EMG and EEG averages.

**Fig. 3:**
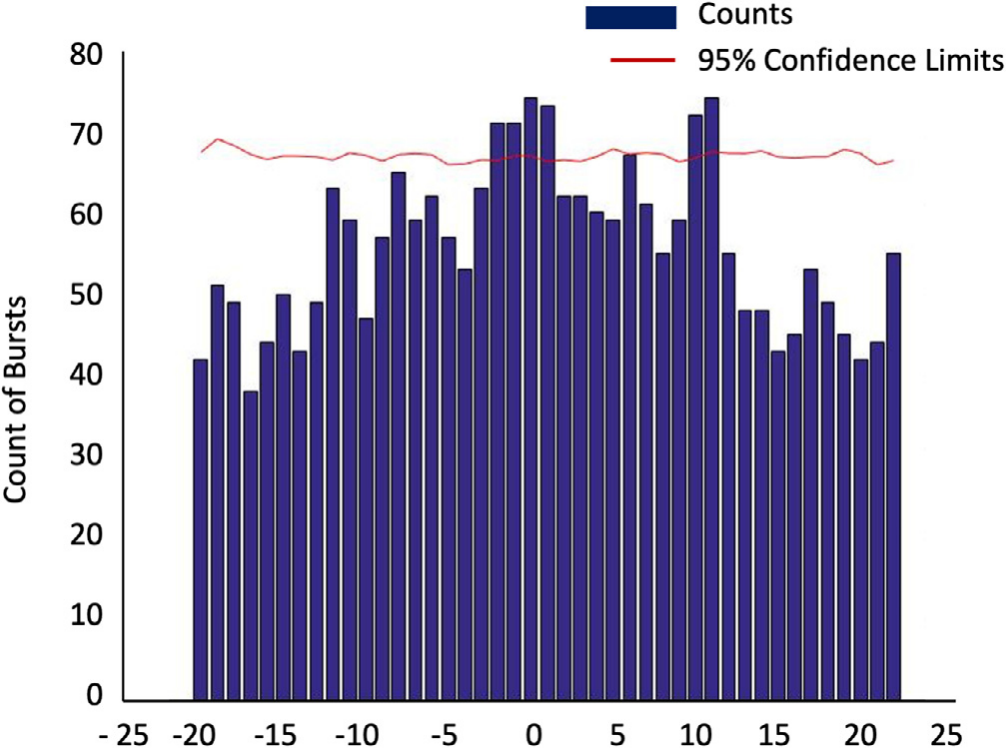
Comparison of myoclonic burst latencies on the left and right triceps from patient 2. Bursts with latencies from −20 to +20 ms were clustered and counted in 1 ms bins. The 95% confidence limit was also calculated.

When comparing the latencies between myoclonic bursts in contralateral homologous muscles, we found that the latencies clustered around 0 ms and +10 ms in patient 1. This clustering was significant, as shown in Fig. 3.

### 3.3 Coherence

#### 3.3.1 Musculo-muscular coherence

**Fig. 4:**
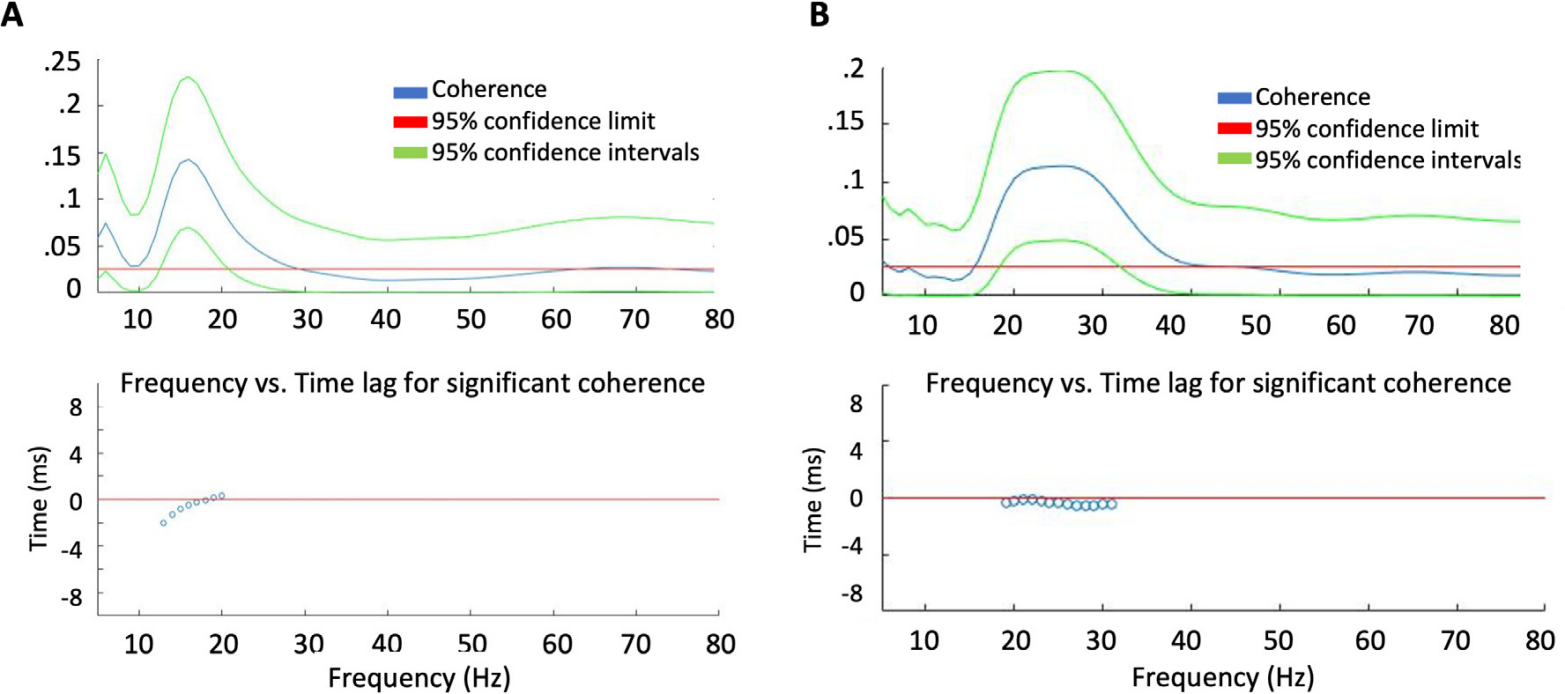
A: Patient 2, The top part shows coherence plotted as a function of frequency between right and left extensor carpi radialis while holding both arms up. The bottom part shows that the time lag in the range of significant coherence is less than 3 ms. B: Patient 1, plots are similar to Part A. Coherence between right and left extensor carpi radialis is near 0 ms time lag.

In both subjects, there was significant coherence in the beta band between antagonist pairs of muscles during the different tasks. There was also coherence between homologous contralateral muscles particularly when both arms were outstretched. At the times with significant coherence, the time lag was very close to 0 ms, as shown in Fig. 4.

## 4 Discussion

With the EMG measurements of homologous contralateral muscles, we were able to confirm our clinical observation that although the myoclonus was multifocal, there were clear episodes in which both sides were bursting in synchrony. This was further supported by significant muscular-muscular coherence between homologous muscles with a coherence phase lag around 0 ms. With the back-average technique and subsequent latency clustering of the myoclonus between homologous muscles, we were able to find a cluster around 0 ms and another one around 10 ms. This is particularly interesting because ∼10 ms is in the range that has been described as the conduction time between contralateral motor cortices across the corpus callosum (Ferbert et al., 1992, Hanajima et al., 2001). This may suggest that the myoclonic activity on one side of the cortex can induce myoclonus on the contralateral cortex. The 10 ms lag between hemispheres has been described previously (Shibasaki et al., 1978, Wilkins et al., 1984). However, this does not explain the cluster around 0 ms, which is more compatible with a common source, which is most likely subcortical. The presence of both a cortical and a reticular source has been described before in patients with post-hypoxic myoclonus (Hallett et al., 1979). Regarding these subcortical sources, another question is if it is activating the descending pathways directly or if it is activating the bilateral cortex simultaneously.

The idea of a subcortical site of origin of the myoclonus had already been proposed in other progressive myoclonic epilepsies (Cantello et al., 1997) but to date not in sialidosis. This is important not only to have a better understanding of the physiopathology of the disease but also thinking about therapeutic approaches as there is medication, such as clonazepam, known to be more effective for the treatment of subcortical myoclonus than other anti-myoclonic medication (Mills and Mari, 2015).

The main limitation of this study is the small number of patients, but it is difficult to enroll a more significant number of patients in such studies given the rarity of these conditions. Also, the patients were taking medication known to reduce myoclonus (see Table 1), but this should not be a confounder concerning our hypothesis of an alternate subcortical source of the myoclonus.

Regarding the localization of the subcortical source, if we consider the work by Huang et al in which they found the blink recovery cycle to be normal in sialidosis patients (Huang et al., 2008), we can infer that there is not a generalized brainstem hyperexcitability. To further localize the origin of the subcortical myoclonus, other tools, such as magnetoencephalography or functional MRI, may be helpful.
